# Henry Ward Beecher

**Published:** 1887-04

**Authors:** 


### HENRY WARD BEECHER.

A great soul has been translated into the realm of spirits ; an immortal spirit has taken its first degree in the order of progression toward the infinite. Not alone does the city of his adoption mourn that Henry Ward Beecher is no longer a visible presence and a power in its midst, but all Christendom is carrying a great burden of sorrow in its heart ; for he whose loss no other is able to supply, was an exemplar of the broadest charity toward all men, without regard to race, condition or order of belief.

As a theologian, looking

"Through nature up to nature's God,"

he refused the limitations of creeds and church tenets, which prohibit freedom of thought, and cramp, as in a mould, the most varied intellects to a common likeness. No other than he, standing, at least nominally, within the Congregational Body, would have been able to hold his outspoken course and retain his footing.

If great in his prosperity, he was no less so in affliction. A very man, he walked above the storm of contending factions on his upward way, and though himself the centre of commotion, from his sublime elevation, he seemed scarcely to heed it. No man that lived could so sway the multitude. In his presence the howling mob was dumb. It was so here in anti-slavery times ; it was no less so abroad when he rose like a wall between the Republic and its assailants. Without assuming to be either a scholar or a logician, as gauged by scholastic rules, he was *great* in everything he undertook, and as a leader of men would have been great anywhere. Brooklyn will long remember his going out as the saddest of its days. More than all, the poor, the lowly, the down-trodden, deplore the loss of one whose heart and hand were always open to them, and never empty.

Such was *Henry Ward Beecher.*

Think of him not as lost, but gone before,
In the near realm,
Whose shining border touches on our shore;
To point the way that finds the open door,
And guide the helm.

That he was good, we know, that he was great,
Knew all the land ;
Yet all that in him lived, in higher state
Is living now, love, joy, compassion ; even hate
Of crushing hand.

No words so sweet, no recompense so dear,
When comes the end,
As whispered accents by the lowly bier,
That tremble upon lips, grievous, sincere :
"*He was my friend.*"

